# Effect of ultrasound on keratin valorization from chicken feather waste: Process optimization and keratin characterization

**DOI:** 10.1016/j.ultsonch.2023.106297

**Published:** 2023-01-10

**Authors:** Xiaojie Qin, Chuan Yang, Yujie Guo, Jiqian Liu, Johannes H. Bitter, Elinor L. Scott, Chunhui Zhang

**Affiliations:** a Institute of Food Science and Technology, Chinese Academy of Agricultural Sciences, Beijing 100193, China; b Biobased Chemistry and Technology, Wageningen University and Research, Wageningen 6700AA, Netherlands; c Xinjiang Taikun Group Co. Ltd, Xinjiang Uygur Autonomous Region, Changji 831100, China

**Keywords:** Feather degradation, Keratin regeneration, Ultrasonic treatment, Keratin solubility

## Abstract

Chicken feather (CF) has been deemed as one of the main poultry byproducts with a large amount produced globally. However, the robust chemical nature of chicken feathers has been limiting in its wide-scale utilization and valorization. The study proposed a strategy of keratin regeneration from chicken feather combining ultrasound and Cysteine (Cys)-reduction for keratin regeneration. First, the ultrasonic effect on feather degradation and keratin properties was systematically explored based on Cys-reduction. Results showed that the feather dissolution was significantly improved by increasing both ultrasonic time and power, and the former had a greater impact on keratin yield. However, the treatment time over 4 h led to a decrease of keratin yield, producing more soluble peptides, > 9.7 % of which were < 0.5 kDa. Meanwhile, prolonging time decreased the thermal stability with weight loss at a lower temperature and amino acids content (e.g., Ser, Pro and Gly) of keratin. Conversely, no remarkable damage in chemical structure and thermal stability of regenerated keratin was observed by only increasing ultrasonic power, while the keratin solubility was notably promoted and reached 745.72 mg·g^−1^ in NaOH (0.1 M) solution (400 W, 4 h). The regenerated keratin under optimal conditions (130 W, 2.7 h, and 15 % of Cys) possessed better solubility while without obvious damage in chemical structure, thermal stability, and amino acids composition. The study illustrated that ultrasound physically improved CF degradation and keratin solubility without nature damage and provided an alternative for keratin regeneration involving no toxic reagent, probably holding promise in the utilization and valorization of feather waste.

## Highlights

• Chicken feather (CF) was efficiently dissolved via Cys-reduction assisted with ultrasound (UCR);
• UCR treatment > 4 h led to obvious low thermal stability and AA loss of keratin;
• Ultrasound physically improved CF degradation and keratin solubility in solvents without nature damage;
• Keratin under optimized condition had good stability and solubility without serious AA loss.

## Graphical abstract

**Figure:**
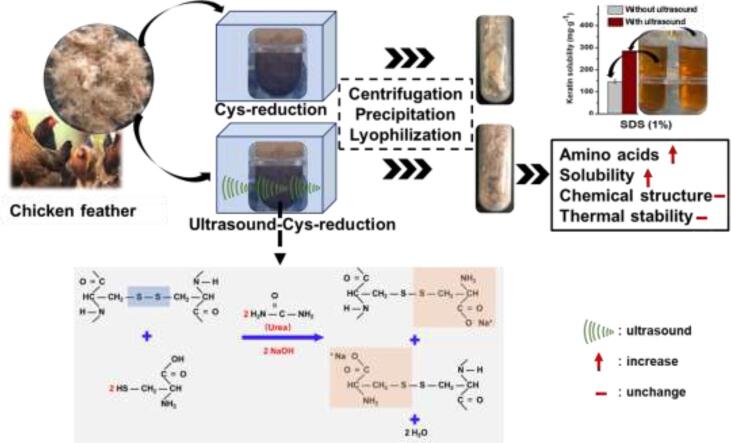

## 1 Introduction

With a great demand for chicken meat and eggs, there are about 50 billion chickens raised globally every year, with a large amount of feather waste produced [1]. Feather waste is a renewable resource containing more than 90 % of keratin [2], rich in Cys, Gly, Pro, and Ser [3]. Feather keratin has potential to be a highly applicable biopolymer with a molecular weight of around 10 500 Da resulting in good biocompatibility, absorbability, biodegradability, and non-toxicity [4], [5]. Some feather waste has been processed into animal feeds, fertilizers, as well as biomaterials, including hydrogels, nanoparticles, sponges, biofilms, and biomedical materials [6], [7], [8], [9], [10], [11]. For example, Wang et al. have recycled chicken feather into keratin nanoparticles and applied them to hemostasis [12]. Despite extensive research devoted to keratin regeneration, the highly stable and resistant nature of feather keratin caused by abundant disulfide cross-link in high content of cystine (7 ∼ 13 %), hydrogen bonds, and hydrophobic interactions has limited its utilization on a large scale [4], [13], [14], and the majority of feather waste end up in landfills leading to both environmental problems and resource waste. Therefore, it is desirable and important to develop an efficient and more eco-friendly way for reusing feather waste into available materials.

There are several ways for keratin regeneration from keratin-rich materials, including oxidation, alkaline hydrolysis, reduction, microwave irradiation, steam explosion, sulfitolysis, ionic liquids, and enzymolysis [15]. Although chemical hydrolysis possesses high efficiency in solubilizing keratin, most of the current methods can produce chemical residues (e.g., sodium dodecyl sulfate, SDS), which limits the application in food and pharmaceutical areas, even with toxic chemicals (e.g., mercaptoethanol, MEC), resulting in environmental problems. Additionally, the methods of oxidation, alkaline, and sufitolysis have been reported to own the risk of damaging protein backbone and amino acid residue (e.g., Trp and Met) [16], [17], [18], while thermal hydrolysis may result in severe degradation and chemical modification though with less requirement of reagents [19]. The methods with microwave, ionic liquids (IL), and enzymes are more eco-friendly compared to other methods, however, exhibit drawbacks as well, such as time-consuming, low yield, and high cost [20], [21]. For instance, the feather-degradation rate only reached about 47.56 % via keratinase enzymolysis after 1 220 min [22], while there was feather keratin with a maximum yield of 21.5 % was recycled through the ionic liquid of 1-hydroxyethyl-3methylimidazolium bis (trifluoromethanesulfonyl) amide ([HOEMIm][NTf2]) after 4 h [23]. Hence, it is a pressing issue to find a more suitable method to regenerate feather keratin with minimal usage of chemicals and with high efficiency at a low cost.

Reduction hydrolysis under alkaline conditions is the most common way for keratin extraction through which the product quality can be controlled via different solvents, reaction time, and other parameters [24], [25]. The use of reducing reagents like thiols has lots of disadvantages, such as being expensive and harmful to both humans and the environment. Xu. et al. have proposed a reduction way using Cys as reducing reagent, and urea as protein denaturant instead of MEC for keratin extraction, with which the disulfide bonds were broken down controllably [26]. The amino acid Cys exhibits strong reducibility owing to the thiol group, and more importantly, it can be commercially produced via fermentation, thus being an environmentally friendly agent [27]. Also, urea could be recycled from extraction solutions showing sustainability [28].

Besides, ultrasound has been widely applied in various areas, such as food, biomedicine, and cosmetics. On the one hand, the mechanical vibration effect can promote the contact between material and medium, thus facilitating the reaction process. On the other hand, destruction often occurs with acoustic cavitation through a sonication medium. Generally, the acoustic streaming of mechanical vibration effect can enhance mass transfer and finally result in productivities in industrials. For example, it has been reported that the yield of collagen Ⅱ (3.37 g) from chicken sternal cartiage was greatly increased by treating with ultrasound for 36 min compared with non-ultrasound group (1.73 g), with an improvement of functional properties, such as foaming and emulsifying properties [29]. In the study of regenerating keratin from feather using (1-butyl-3-methylimidazolium chloride, [BMIM]Cl) assisted with ultrasound, ultrasonic irradiation significantly improved keratin dissolution compared with the conventional method and shortened process time from 2 h to less than 20 min [21]. Taken together, it is a promising way to combine ultrasound and Cys reduction for keratin extraction.

The main purpose of this study was to develop an efficient and sustainable process combining Cys-reduction and ultrasound for feather waste valorization. Firstly, the effect of ultrasonic power and time on the yield and physical properties (e.g., feather dissolubility, keratin yield, chemical structure, crystallinity) of regenerated keratin was investigated based on the Cys-reduction method. Then, the process optimization of Cys-reduction assisted with ultrasound was conducted using Box-Behnken Design. Finally, feather keratin was prepared under optimal conditions, the physicochemical characterization, including chemical structure, crystallinity, thermal stability, amino acids composition, and keratin solubility, of which was also assayed taking keratin untreated with ultrasound as control. The study systematically explored the potential application of ultrasound in feather degradation and might be meaningful in providing an alternative strategy for utilizing feather waste.

## 2 Material and methods

### 2.1 Material and chemicals

Chicken feather was supplied by the Institute of Animal Sciences of CAAS. Feathers were cleaned with detergent and distilled water and then were dried at 50 °C in an oven for 48 h. Subsequently, the dried feathers were milled into a cotton shape for keratin extraction. Reagents including urea, Cys, etc. were of analytical grade and purchased from Sinopharm Chemical Reagent Co., Ltd (China). HPLC grade acetonitrile (ACN) was supplied by Thermo Fisher Scientific (Waltham, USA). The deionized water was obtained via Milli-Q 50 system (Millipore Corp., Milford, MA, USA).

### 2.2 Keratin extraction using Cys reduction assisted with ultrasound

2 g of milled feather were mixed with 20 mL of a solution containing 8 M urea and 15 % Cys, which has been adjusted to pH 10.5. The mixture was treated with ultrasound at various power (from 100 W to 600 W) for varying times (2 h to 10 h) at 70 °C using an ultrasonic instrument at the frequency of 40 kHz (YQ1001C, Yijing Ultrasonic Instrument Co., Ltd, Shanghai, China). Then, the mixture was centrifuged at 8 000 rpm for 20 min thus obtaining supernatant. The keratin was further precipitated by adjusting the supernatant to pH 4 with hydrochloric acid and then was washed three times with distilled water under centrifugation of 8 000 rpm for 20 min. Finally, the regenerated keratin was lyophilized and weighed. The effect of both ultrasonic power and time on keratin yield was analyzed by linear regression modeling. The yield (Y) of regenerated keratin was calculated with Eq. (1):

(1) $$Y\left(\%\right)\;=\;\frac{m_{0}}{m_{1}}\times 100$$

where *m*_0_ represented the weight of dried keratin, and *m*_1_ represented the weight of introduced chicken feather on a dry base.

### 2.3 The morphology of chicken feather

The uncrushed feathers were treated by ultrasound assisted Cys-reduction under conditions as part 2.2 described for various times (2 to 10 h) with ultrasonic power of 200 W. The treated feathers were washed three times with distilled water, and then were stored in 4 % paraformaldehyde at 4 °C. The feathers were observed using an inverted microscope (Zeiss Axio Vert.A1, Zeiss, Germany) at 2 ∼ 3 cm from the thick end with a magnification of ×100.

### 2.4 Characterization of regenerated keratin

The keratin extracted under conditions as part 2.2 described (200 W for 2 to 10 h and 100 to 600 W for 4 h) was characterized with raw chicken feather as control. For chemical structure, the samples were determined referring to previous study [30]. Keratin samples were ground with potassium bromide (KBr) under dried air, and then the mixture was pressed into a 1 mm pellet and scanned in a frequency from 4 000 to 400 cm^−1^ using Fourier transform infrared spectroscopy (FTIR) spectrometer (Model Nicolet Nexus 470, Thermo Fisher Scientific, Waltham, MA, USA).

For thermogravimetric analysis (TGA) and derivative thermogravimetric analysis (DTG), the samples were analyzed via Thermogravimetric Analyzer (Pyris Diamond TG/DTA, PerkinElmer, USA) in a flowing nitrogen atmosphere. The samples were loaded in aluminum pans and equilibrated, and then were heated from 30 °C to 500 °C with a heating scan rate of 10 °C·min^−1^.

For crystallinity analysis, X-ray diffraction (XRD) study was performed using a Panalytical Empyrean powder diffractometer (Ultima IV, Rigaku, Japan) at 25 °C. The CuKα1 radiation (λ = 1.54 Å) was produced at 40 kV and 40 mA. Diffraction intensities were recorded with 2θ ranging from 5° to 80° with a step size of 0.02° 2θ at a scan speed of 5°/min. The crystallinity index (CI) was calculated with Eq. (2) [31]:

(2) $$\mathrm{CI}\;\left(\%\right)=\frac{A_{\mathrm{crystal}}}{A_{\mathrm{total}}}\times 100$$

where A_total_ is the total area under the diffraction curve from 5 to 60°, and A_cystal_ is the area below the crystal diffraction peaks.

### 2.5 Content of soluble protein and peptides

2 g of milled feather mixed with 20 mL of the buffer as part 2.2 mentioned were treated with ultrasound at 200 W for different times (2, 4, 6, 8, and 10 h) and with varying ultrasound (100 to 600 W) at 70 °C. The supernatant was obtained by centrifuging the mixture at 8 000 rpm for 20 min and then was diluted 100 times using distilled water for assaying soluble protein and peptides according to the method as Lowry et al. mentioned [32], using bovine serum albumin as a standard.

For soluble protein determining, briefly, 20 μL of the diluted supernatant and 200 μL of reagents were added into 96-well plates, and the mixture was then stood for 30 min at 37 °C. After that, the absorbance was detected via a microplate reader (Synergy H1, BioTek, USA) at 660 nm.

In order to determine soluble peptides, 0.25 mL of diluted supernatant was fully mixed with an equal volume of trichloroacetic acid (TCA) solution (10 %) and stood for 60 min at room temperature. After that, the final supernatant was prepared by centrifuging the mixture at 10 000 × g for 20 min. Subsequently, the procedure for peptides detection was the same as that of protein measurement above.

### 2.6 Distribution of molecular weight of peptides

The molecular weight (M_w_) of soluble peptides in extraction supernatant obtained as part 2.5 described was assayed using Agilent liquid chromatograph 1 200 with a UV detector (Agilent, CA, USA) [33]. The TSK gel filtration column (G2000 SWXL 300 mm × 7.8 mm, Tosoh Co., Tokyo, Japan) was adopted, and the mobile phase was composed of water/acetonitrile/trifluoroacetic acid (55/45/0.1, v/v/v). The flow rate of the mobile phase was 0.5 mL/min with column thermo-stated at 40 °C. The injection volume of the sample was 10 μL, and the absorbance was monitored at 220 nm. The standards used for M_W_ calibration curve included Gly-Sar (146.15 Da), tetrapeptide Gly-Gly-Tyr-Arg (451 Da), bacitracin (1 423 Da), aprotinin (6 511 Da), cytochrome *C* (12 355 Da) (Sigma Aldrich, St. Louis, MO, USA). The data were analyzed via gel permeation chromatography software.

### 2.7 Amino acids analysis

The amino acids composition in regenerated keratin were analyzed using peroxidation and acid hydrolysis method [34]. Briefly, the accurately weighted samples (∼10 mg) were slowly dispersed in 2 mL ice-cold performic acid solution (hydrogen peroxide: formic acid = 1:9) which has been freshly prepared. The mixture was incubated in fridge for 16 h, and then 0.3 mL ice-cold hydrobromic acid was added and stood for another 30 min on ice. The mixture was dried with nitrogen blowing device at 50 °C, after which 10 mL of HCl (6 M) containing 0.1 % phenol were added for 24-h hydrolysis at 110 °C. The hydrolysate was filtered and transferred into a volumetric flask (50 mL). After that, 1 mL of filtered hydrolysate was dried via nitrogen blowing device at 37 °C and then redissolved with 1 mL of HCl (0.02 M). The sample solution was further filtrated through 0.22 μm membrane, and pre-derivatized with OPA before HPLC (Thermo Fisher Ultrimate 3000, USA) analysis. The method can determine most amino acids except for Tyr and Trp. Especially, Cys could be quantified via oxidizing into cysteic acid.

### 2.8 Optimization of keratin extraction via Box-Behnken experiment

For the single-factor experiment, urea (8 M), pH (10.5), and temperature (70 °C) were constant in the three groups, while the “Ultrasonic time,” “Ultrasonic power,” “Solid-liquid ratio,” and “Cys fraction” were separately served as the variation in three control groups, with the other three factors serving as a fixed factor. Briefly, the milled chicken feather was firstly fully mixed with buffer containing 8 M urea and various fractions of Cys (5, 10, 15, 20, 25 %) at a solid–liquid ratio of 1: 10 to 1:30, the pH of which was adjusted to 10.5 using NaOH (6 M). Then, the mixtures were treated with ultrasound at varying powers (100, 200, 300, 400, 500, 600 W) for a certain time (2, 4, 6, 8, 10 h). After that, the mixtures were centrifuged at 8 000 rpm for 20 min to precipitate undissolved residues. The supernatant was adjusted to pH 4 to precipitate keratin. After washing three times with distilled water, the regenerated keratin was freeze-dried and weighed to calculate the yield using Eq. (1).

The Box-Behnken experiment was designed based on the effect of three independent variables of “Ultrasonic time,” “Ultrasonic power,” and “Cys fraction” on keratin yield, and three levels of each factor were shown in Table S1. The “Solid-liquid ratio” was kept at 1:10 because a lower ratio led to less keratin due to the buoyancy effect. The results were analyzed by multiple regression fitting analysis using Design Expert 8.0 software. Finally, the verification test was conducted under optimized conditions (130 W, 2.7 h, 15 % Cys). Analysis of FTIR, TGA, XRD, and amino acids composition of keratin from verification test was conducted, taking the keratin extracted without ultrasonic treatment (2.7 h, 15 % Cys) as control. The methods were the same as those in part 2.4 and 2.7.

### 2.9 Keratin solubility in different solvents

The solubility of regenerated keratin assisted with or without ultrasound in different solvents was investigated, referring to a previous study with some modifications [35]. In this study, four kinds of solvents were prepared: SDS (1 %), NaOH (0.1 M), Na_3_PO_4_ (0.01 M, pH 7.5) and Na_3_PO_4_ (0.01 M, pH 7.5, 2 % urea). Briefly, 100 mg of samples were dispersed in 5 mL of various solvents and incubated at room temperature for 60 min. Then, the mixtures were centrifuged at 3 000 × g for 15 min. The supernatant was diluted 10 times to detect the soluble protein content using the method as part 2.5 described.

### 2.10 Statistical analysis

Statistical analysis was conducted via SPSS 17.0 software. The figures were made by Origin8.5 software. Differences among mean values were established using the Duncan multiple range test at *P* < 0.05. All analyses were carried out in triplicate samples, and the results were presented as mean ± standard deviation (SD).

## 3 Result and discussion

### 3.1 Effect of ultrasound on feather degradation

#### 3.1.1 Feather dissolubility and keratin yield treated with ultrasound

**Fig. 1:**
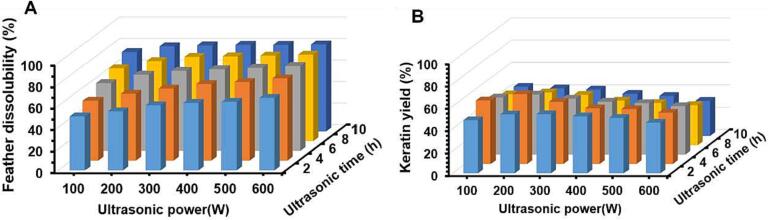
(A) Feather dissolubility (%), and (B) keratin yield (%) from chicken feather treated with various ultrasonic power for different time. The ultrasonic power changed from 100 to 600 W, and the time was from 2 to 10 h. The result was present as a mean value, n = 3. The linear regression model of keratin yield was Y = 63.736–0.02X_power_-1.67X_time_ (R^2^ = 0.751, *P* < 0.001).

This part was conducted to explore the effect of varying ultrasonic power (100 ∼ 600 W) and time (2 ∼ 10 h) on both feather dissolubility and keratin yield based on the Cys reduction method. Chicken feather was firstly swelled by urea, in light of which keratin dissolution was processed by disulfide bonds breakage with the thiol group consisting in Cys. Fig. 1A showed that feathers were dissolved sharply when the ultrasonic time increased from 2 h to 4 h at low ultrasonic power (e.g., 100 and 200 W), and then were gradually promoted with the increase of both ultrasonic power and time. The feather dissolubility reached about 79.1 % and 80.7 % at 200 W-10 h and 600 W-10 h, respectively, exhibiting no significant difference (*P* > 0.05). There was about 20 % of feather undissolved, possibly resulting from the floating on buffer surface. Fig. 1B showed that keratin yield increased at first and then fell with time and power increasing, with the maximum value of 62.65 % under the condition of 200 W–4 h. Ultrasound might facilitate the production of free amino acids and peptides with small Mw with the combined effect of mechanical vibration and acoustic cavitation, and stronger ultrasound might result in over-degradation of keratin [21], [36]. Despite a relatively lower keratin yield compared to previous methods where MEC, thiourea, and thioglycolic acid were adopted as reducing agents (with keratin yield of 67 ∼ 91 %) [15], the present study avoided the use and residue of toxic chemicals while taking a shorter time. The potential of the Cys reduction method assisted with ultrasound in solubilizing feather waste is still worthy of exploring. Through linear regression analysis, ultrasonic time was found to be more influential on keratin yield, while ultrasonic power had less influence (Fig. 1B). The feather morphology, soluble protein composition, and physicochemical properties of extracted keratin would be further determined to investigate the effect of ultrasound and Cys reduction on chicken feather.

#### 3.1.2 Morphological changes of feather treated by ultrasound

**Fig. 2:**
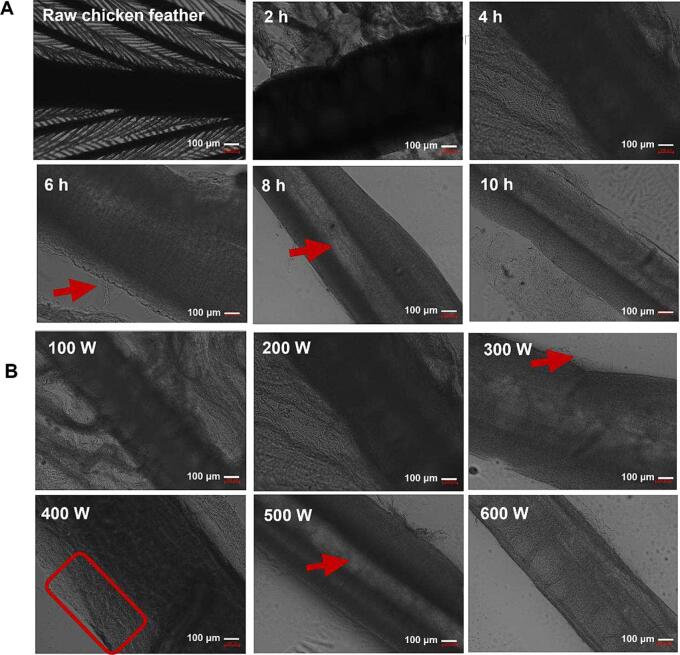
(A) The apparent structure of chicken feather treated without or with ultrasound at 200 W for various time (from 2 to 10 h), and (B) treated with varying ultrasonic power (from 100 to 600 W) for 4 h. The feather were photographed at 2 ∼ 3 cm from the thick end with a magnification of × 100.

The effect of various ultrasonic power and time on feather morphology was visually assayed by treating unmilling feather with ultrasound for a certain time. Fig. 2 showed that the barb ridges of feather were obviously dissolved with time and power increasing. The feather branches were totally solubilized when treated at 200 W for 6 h and 300 W for 4 h, indicating a positive effect of ultrasound. The feather shaft was significantly broken with a rough surface (e.g., 400 W-4 h), possibly caused by the combined action of ultrasound and reagents. The shaft was thinner with a hollow part that clearly emerged when the process lasted for 8 h at 200 W and increased to 500 W for 4 h. Unlike the transverse cracks and cavities on the surface given by steam explosion treatment [19], the feather fibers were longitudinally dissolved layer by layer with ultrasonic treatment. In general, ultrasound could positively improve feather dissolution.

#### 3.1.3 Analysis of soluble peptides in feather hydrolysate

**Fig. 3:**
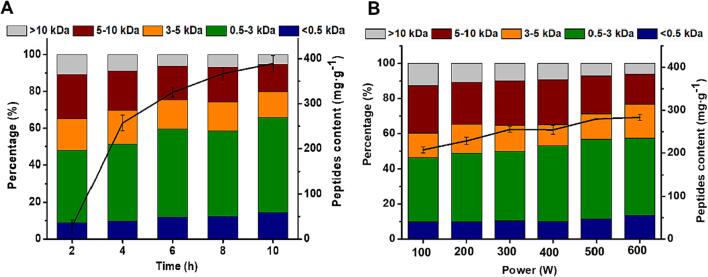
(A) Effect of various ultrasonic time (2 ∼ 10 h, 200 W) and (B) ultrasonic power (100 ∼ 600 W, 4 h) on the content and Mw distribution of soluble peptides in extracting solution. The values were present as mean ± SD, n = 3.

The effect of different ultrasonic time and power on feather hydrolysis was evaluated by assaying soluble peptides (Fig. 3A and B). The result showed that ultrasonic time remarkably improved peptides content growing from about 29 mg·mL^−1^ at 2 h to 389 mg·mL^−1^ at10 h, while ultrasonic power made less effect with peptides content growing from about 207 mg·mL^−1^ at 100 W to 283 mg·mL^−1^ at 600 W for 4 h. Fig. 3A indicated that there was about 47 % of them with Mw less than 3 kDa at 2 h while the peptides < 0.5 kDa was about 8.7 %. As processing time was prolonged to 10 h, around 65 % of peptides < 3 kDa were produced, among which about 20 % were smaller than 0.5 kDa. Similarly, power rising also resulted in the proportion increasement of smaller peptides, which contributed to feather dissolution (Fig. 3B). The result illustrated that this process effectively facilitated the breakage of feather keratin. Despite the beneficial effect of ultrasound on feather degradation, the quality of regenerated keratin should also be concerned. The influence of various ultrasonic power and time on keratin properties solubility and amino acids composition will be further discussed.

### 3.2 Characterization of regenerated keratin treated by ultrasound

#### 3.2.1 Chemical structure analysis

**Fig. 4:**
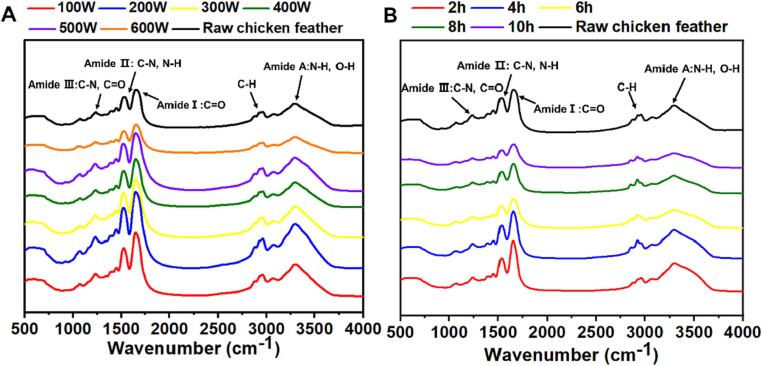
FTIR spectrogram of regenerated feather keratin treated for 2 to 10 h at 200 W (A) and treated from 100 to 600 W for 4 h (B).

FTIR measurement was used to assess the effect of ultrasound on structural changes in keratin. The FTIR spectrum indicated that various regenerated keratin exhibited similar bands compared to raw chicken feather (Fig. 4A and B). The absorption bands at about 3 292 cm^−1^ were observed known as the Amide A vibrations regarding the N—H stretching [37], and the peak at around 2 932 cm^−1^ was related to the asymmetrical stretch of CH_2_, CH_3_, and C—H [38], [39]. The strong bands that appeared at 1 657 cm^−1^ mainly resulted from the C=O stretching of Amide I in α-helix structures, while the peak at 1 535 cm^−1^ assigned to the C—N stretching of Amide II coupled with N—H bending vibration [29]. At about 1 396 cm^−1^ and 1 234 cm^−1^, the bands were mainly related to C—N and C—C stretching, N—H plane bending, and C=O bending vibration of Amide III [21]. As result presented, the increase of ultrasonic power and treating time would not distinctly change the characteristic chemical groups, or produce new functional groups in regenerated keratin.

#### 3.2.2 Thermal stability analysis

**Fig. 5:**
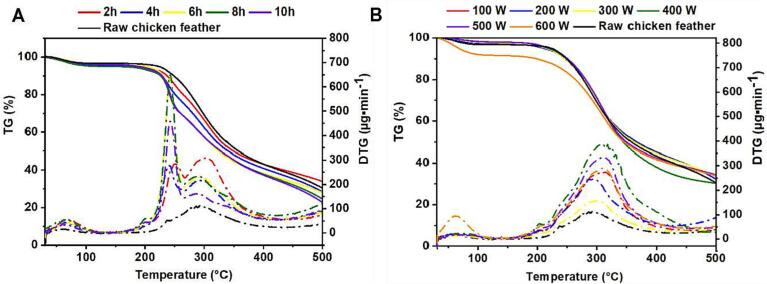
TGA traces of regenerated feather keratin treated for 2 to 10 h at 200 W (A) and treated from 100 to 600 W for 4 h (B).

The effect of ultrasound on the thermal stability of feather keratin was assayed by TGA. Fig. 5A and B showed that treating time was found to have an obvious influence on keratin weight loss instead of ultrasonic power compared to raw chicken feather. Raw chicken feather exhibited two phases of weight loss, while regenerated keratin treated over 6 h lost weight in three steps. The result showed that there were about 5.4 % of weight loss happened in raw chicken feather at 100.32 °C, and the keratin treated with ultrasound for 2 to 10 h had weight loss of 3.2 ∼ 4.6 %. This step was caused by moisture evaporation, and the difference between raw chicken feather and extracted keratin was mainly due to different moisture content [40]. Subsequently, the weight of raw chicken feather dropped to 39.4 % from 200 to 400 °C due to the escape of hydrogen sulfides and sulfur dioxides resulting from disulfide bonds damage [41], [42], [43]. Similarly, the second weight loss of extracted keratin happened when heating to 180 °C around, followed by a further loss at about 260 °C. For regenerated keratin, the second phase was possibly attributed to the breakage of α-keratin fibers and smaller particle size, while the final weight loss was induced by the deconstructing of β-sheet consisting of disulfide bonds and polypeptide chains, and larger particle size [40], [44]. Importantly, longer ultrasonic time led to a sharper decline of keratin weight in the second phase, specially treated for over 6 h. Meanwhile, the keratin treated with longer-time ultrasound had weight loss at a lower temperature. It indicated that long-time ultrasound potentially promoted the cleavage of keratin fibers and reduced disulfide-bond content, thus leading to relatively poorer thermal stability. When changing ultrasonic power, no significant difference was found between various samples, except for keratin at 600 W losing weight remarkably which might result from higher bonding moisture. The result indicated that the thermal stability of regenerated keratin might be potentially reduced by combined action of Cys and ultrasound for over 6 h, while solely improving ultrasonic power showed no obvious effect.

#### 3.2.3 Crystallinity analysis

**Fig. 6:**
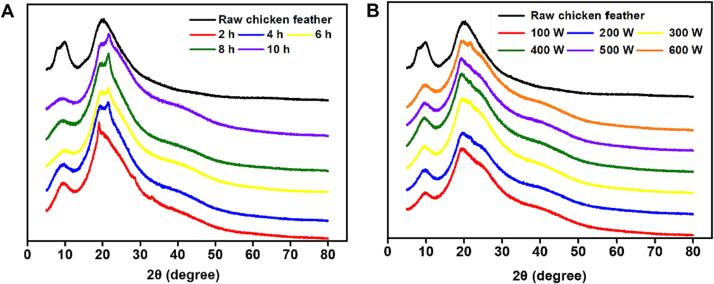
X-ray diffraction spectrogram of regenerated feather keratin treated for 2 to 10 h at 200 W (A) and treated from 100 to 600 W for 4 h (B).

Fig. 6A and B presented the XRD result of raw chicken feather and regenerated keratin to evaluate their secondary structure and crystallinity. Similar to the previous study, two notable 2θ peaks appeared at about 9.8° and 20° for each keratin material [12], [22]. The 2θ peak at about 9.8° was related to both α-helix and β-sheet, while 20° was also a typical peak value of β-sheet structure [45]. Compared to raw chicken feather, regenerated keratin exhibited lower intensity of peak at about 9.8°. It indicated that the combined treatment of Cys and ultrasound might deconstruct α-helix and β-sheet structure in raw chicken feather resulting in a decreasing crystallinity of regenerated keratin. Nevertheless, the peak at about 9.8° became smaller with ultrasonic time rising while showing no notable changes with the power increase. These changes were consistent with the result of thermal stability, where there was a sharp decline in the second weight loss phase. Additionally, broader peaks at about 20° were observed in ultrasound-treated samples, which might be attributed to the overlap of the β-sheet and β-turn peaks [21]. Keratin has been reported to own a CI of about 63.6 %, with a mixture of crystal and amorphous structure [46], [47]. Some treatments have been found to remarkably change the keratin CI. For example, the keratin extracted using 2 ME had a CI of 30.46 % [31]. In this study, the regenerated keratin showed a decrease in crystallinity rate at about 62 % compared to raw chicken feather (66.27 %), and different conditions made no remarkable changes in CI (Table S2).

Overall, ultrasound facilitated feather dissolution based on the Cys reduction method without changing the main chemical structure of recycled keratin. However, the thermal stability of recycled keratin would be reduced by long-time treatment (> 6h). In our study, the solubility of regenerated keratin in different solvents would be further explored, which was closely related to chemical structure and crystallinity [35].

#### 3.2.4 Keratin solubility in different solvents

**Fig. 7:**
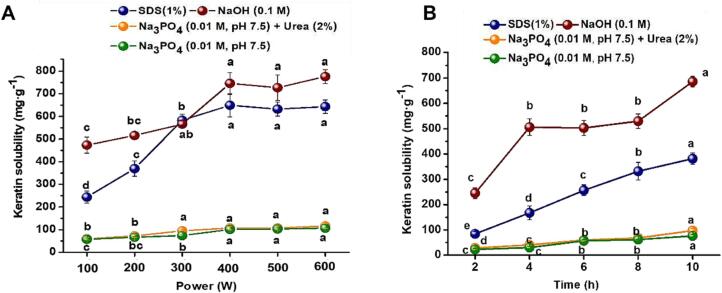
(A) Effect of various ultrasonic time (2 ∼ 10 h, 200 W) and (B) ultrasonic power (100 ∼ 600 W, 4 h) on the solubility of extracted keratin in different solvents. The values were present as mean ± SD, n = 3. The lowercase letters “a ∼ e” indicated the significant difference in soluble protein content between different ultrasonic conditions (*P* < 0.05).

Abundant covalent and non-covalent bonds have resulted in poor solubility of feather keratin. However, proper solubility is the prerequisite for further utilization of keratin widely. The effect of ultrasound on keratin solubility in various solvents were studied. Fig. 7 indicated that 0.1 M NaOH owned the best ability in dissolving keratin, followed by 1 % SDS solution, while the solutions of Na_3_PO_4_ (0.01 M, pH7.5) and Na_3_PO_4_ (0.01 M, pH7.5, 2 % urea) dissolved keratin limitedly. Generally, the exposed hydrophobic residues during denaturation are considered as the major reason of protein aggregation [48], and the protein solubility will be increased when the electrostatic repulsion is dominant compared to the hydrophobic interaction [48], [49]. In this study, the high pH value was crucial to keratin solubility by changing the electrostatic interactions, and SDS presented a good capacity of dissolving keratin by disrupting the hydrophobic interactions. Nevertheless, urea did not obviously improve keratin dissolution, indicating a relatively weak force from hydrogen bonds in recycled keratin. Additionally, it should be noted that keratin solubility showed a growing trend with the increasing of ultrasonic time and power, possibly caused by the physical changes of intermolecular crosslinks. Previously, it has also been concluded that sonication mainly produced a physical effect on keratin with minimal effect on the chemical structure, which would not affect the backbone structure [21]. In our case, the increasing ultrasonic intensity likely weakened the degree of physical crosslinking of recycled keratin, and thus improved its solubility in aqueous solutions.

#### 3.2.5 Analysis of amino acids in regenerated keratin

**Fig. 8:**
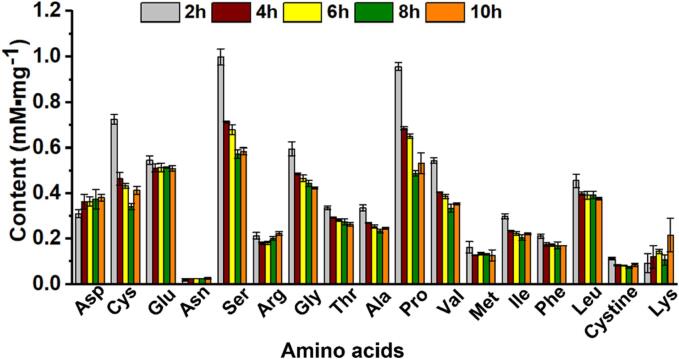
Content of 17 kinds of amino acids in extracted keratin treated for 2 to 10 h. The values were present as mean ± SD, n = 3.

Considering the significant effect of treating time on the thermal stability and solubility of recycled keratin, the amino acids composition was assayed which might be a key factor. The result showed that most amino acids became less with treating time increasing from 4 to 10 h, such as Gly, Ala, Ser, Pro, Val, Ile, Phe, and so on (Fig. 8). Long-time treatment resulted in a high hydrolysis degree and serious amino acids loss. Also, the cystine content presented a decrease with time prolonging, which might be due to the growing reduction degree via Cys. As reported, cystine was quite sensitive in alkaline solutions [15], the disulfide bonds of which were easily cut with the presence of sulfhydryl. However, longer processing time also led to an obvious Cys loss. Conversely, an increase of Asp and Lys was observed when prolonging treating time. Similar to previous study, serious loss of Thr, Ser, and Cys was found when treating feather with 0.1 M NaOH at 90 °C, while Lys increased [50]. The amino acids composition change resulted from long-time processing might closely relate to the keratin solubility, and the process time should be controlled to avoid keratin over-degradation.

Taken together, Cys reduction assisted with ultrasound was an effective way to dissolve feather waste. Ultrasonic assistance could potentially improve keratin recycling without obvious chemical deconstruction. However, a long processing time would result in decreased thermal stability and serious loss of amino acids from recycling keratin. Therefore, it is still necessary to find an optimal condition that can endow recycled keratin with good stability and solubility without over-degradation while consuming less energy and time.

### 3.3 Optimization of keratin extraction via Box-Behnken design

#### 3.3.1 Single-factor experiment and Box-Behnken design

**Fig. 9:**
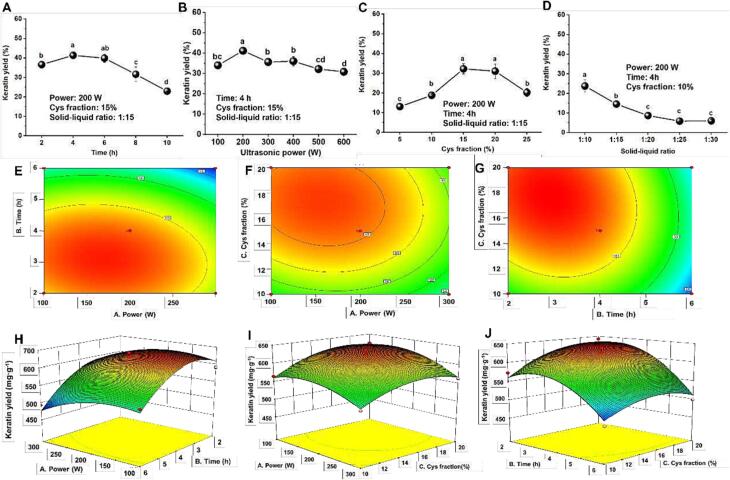
(A) Effect of different ultrasonic time, (B) ultrasonic power, (C) Cys fraction, and (D) ratio of feather to buffer on keratin yield. (E ∼ G) contour plots, and (H ∼ J) response surface plots (3D) showing the effect of various ultrasonic time, power, and Cys fraction on keratin yield. The values were present as mean ± SD, n = 3. Different lowercase letters (a ∼ d) indicate a significant difference (*P <* 0.05) among the samples under different conditions.

Effect of factors including ultrasonic time, ultrasonic power, Cys fraction, and the ratio of feather to buffer on keratin yield were preliminarily investigated (Fig. 9A ∼ D). By treating feather with varying ultrasonic power and time, the extraction yields showed peak values at 200 W and 4 h, respectively, which were significantly higher than others (*P <* 0.05) (Fig. 9A and B). Higher ultrasonic power and longer treating time might lead to excessive degradation of keratin, producing more smaller peptides which has been discussed in part 3.1.3. Fig. 9C indicated that the keratin yield reached the highest point when adding 15 % of Cys (*P <* 0.05). The decrease of keratin yield with higher Cys fraction might be caused by buffer dilution since more NaOH solution was needed for pH adjusting with increasing Cys. Due to the buoyancy of feather on the buffer surface, a high solid–liquid ratio was found to result in less degradation (Fig. 9D). Therefore, the ratio of 1:10 was chosen as constant viable in process optimization. Finally, the optimal conditions of 200 W, 4 h, and 15 % were chosen as central points for the Box-Behnken design.

Seventeen groups of trials were designed for optimizing keratin extraction based on three variables, and the results were analyzed by multiple regression fitting analysis using Design Expert 8.0 software (Table S3). A quadratic regression equation model was obtained as follows: R_1_ = 623.50 − 19.36*A − 46.76*B + 22.18*C − 6.64*AB − 5.95*AC − 11.71*BC − 25.55*A^2^ − 48.55*B^2^ − 27.20*C^2^. The result showed that the model coefficient (R^2^) was 0.9585 with significant deference (*P* < 0.001), and the lack of fit was with an insignificant difference (*P* > 0.0688), indicating that this model fitted well and correctly reflected the relationship of three factors and keratin extraction yield (Table S3). Generally, the effect of different factors on response value can be reflected by contour density and response surface slope. In the present study, ultrasonic time was found to be the most important factor affecting keratin extraction, followed by Cys fraction and ultrasonic power in sequence, which was also consistent with the ANOVA result (*P* < 0.001) (Fig. 9E ∼ J and Table S3). In order to achieve a maximum keratin yield with low energy and time-consumption, the optimal conditions were screened and finally determined as 132.40 W, 2.71 h, and 15.07 % of Cys, under which the theoretical yield was 632.537 mg/g. Considering the practical operability, 130 W, 2.7 h, and 15 % were chosen as the optimal conditions to extract keratin. By verification, a final keratin yield of 640.27 ± 9.50 mg/g was obtained, the relative error of which was 1.22 %. The quality of regenerated keratin would be further explored compared to the keratin without ultrasonic disposal.

#### 3.3.2 Analysis of chemical structure, thermal stability, and crystallinity of regenerated keratin under optimal conditions

**Fig. 10:**
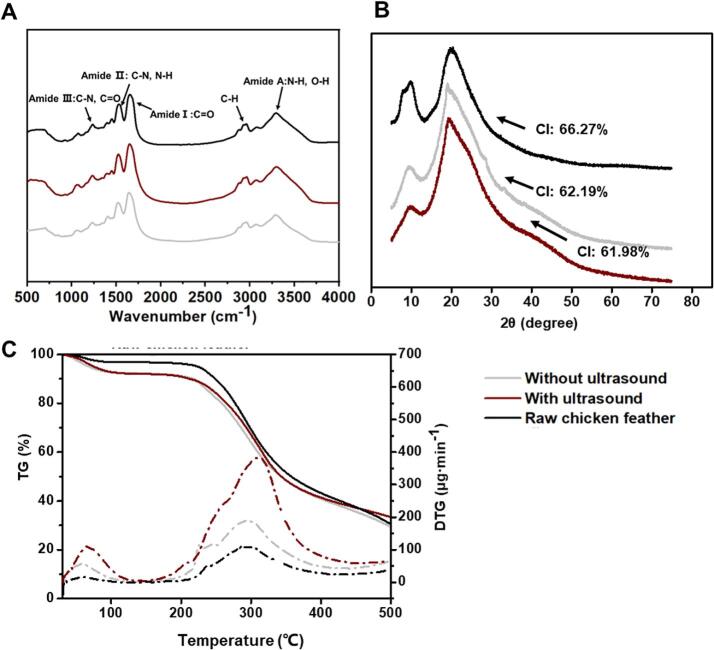
(A) FT-IR spectrogram, (B) X-ray diffraction spectrogram, (C) TGA and DTG traces. The keratin treated with ultrasound was regenerated under optimized conditions (130 W, 2.7 h, 15 % Cys, 70 °C, pH 10.5, 8 M urea, 1:10 solid–liquid ratio), while the other was obtained under the same conditions except for ultrasonic treatment.

The physical properties of regenerated keratin via the ultrasound-Cys-reduction method were analyzed, taking keratin obtained via Cys-reduction and raw chicken feather as control groups. Fig. 10A showed no significant difference between three groups in main chemical groups, including Amide Ⅰ, Ⅱ, III, A, and B. The process within 2.7 h did no remarkable changes in main chemical bonds. By further analyzing the XRD spectrum, both recycled keratins with and without ultrasound exhibited lower intensity of 2θ peak at about 9.8° than the raw chicken feather, indicating lower content of tightly packed crystals (Fig. 10B). The CI of ultrasound-treated and ultrasound-untreated keratin were 61.98 % and 62.19 %, respectively, indicating no obvious difference in crystallinity. For thermal stability, three kinds of keratin materials presented two main steps of weight loss from about 40 ∼ 100 °C and 200 ∼ 400 °C, respectively, attributing to the evaporation of bonding moisture and the rupture of both helical conformation and disulfide bonds [21] (Fig. 10C). The result further proved that ultrasonic assistance would not significantly affect the chemical structure, crystallinity, and thermal stability of recycled feather keratin, and the optimal conditions were feasible for keratin generation.

#### 3.3.3 Analysis of amino acids and solubility in different solvents

**Fig. 11:**
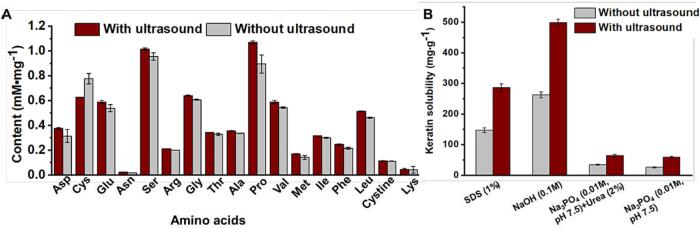
(A) Content of 17 kinds of amino acids and (B) keratin solubility in various solvents. The keratin treated with ultrasound was regenerated under optimized conditions (130 W, 2.7 h, 15 % Cys, 70 °C, pH 10.5, 8 M urea, 1:10 solid–liquid ratio), while the other was obtained under the same conditions except for ultrasonic treatment. The values were present as mean ± SD, n = 3.

The effect of ultrasonic treatment under optimized condition on amino acids composition of keratin was investigated compared to the ultrasound-untreated group (Fig. 11A). The result showed that most of amino acids were richer in ultrasound-treated keratin than ultrasound-untreated group, such as Ser, Gly, Ala, Pro, Val, Phe, and Leu. Different form the serious amino acids loss at higher temperature (> 100 °C), 75 °C treatment would not result in keratin over-degradation [51]. In this study, the ultrasound-treatment improved the total amino acids content (7.45 mM/mg) which was significantly higher than the control group (6.81 mM/mg) (*P* > 0.05). However, ultrasound has resulted in a lower Cys content in recycled keratin while no significant difference was found in cystine content. It indicated that Cys was more susceptible and easily degraded under ultrasonic condition. Consistent with previous studies, the regenerated keratin in the present study contained abundant Pro, Ser, Glu, Val, and Leu [12], [35].

Similar to the result in part 3.2.4, NaOH solution owned the best capacity of dissolving regenerated keratin, which was followed by 1 % SDS solution, while there was less keratin dissolved in Na_3_PO_4_ solution with or without urea at pH of 7.5 (Fig. 11B), further suggesting the strong hydrophobic and electrostatic interactions in keratin and relatively weaker hydrogen bonding. Obviously, the ultrasound-treated keratin possessed higher solubility in various aqueous solutions than the ultrasound-untreated group. Considering no significant difference in chemical structure, crystallinity, and thermal stability has been found between those two groups, it was further verified that ultrasound potentially reduced non-covalent bonds, such as hydrogen bonds, hydrophobic interaction, and Van der Waals force, thus leading to better solubility of recycled keratin.

Taken together, the optimal conditions of 130 W, 2.7 h, and 15 % of Cys were practical for keratin regeneration from feather waste, under which the product reserved complete chemical structure while possessing good stability and solubility. The process realized feather recycling in time-saving way without toxic chemicals, which might be promising in reusing keratin materials in various areas.

## 4 Conclusion

To conclude, ultrasound could improve feather degradation based on the Cys reduction method by physically destroying crosslinking interactions while still retaining the protein backbone structure. However, the long processing time resulted in feather over-degradation producing a large number of peptides with small molecular weight, and the keratin product exhibited low thermal stability and serious amino acids loss. Under the optimal conditions (130 W, 2.7 h, and 15 % Cys) obtained via Box-Behnken design, keratin product exhibited better solubility in various solvents than the control group, while without serious amino acids loss and obvious damage on chemical structure and thermal stability. The optimal process reserved the structural integrity of keratin and endowed it with good thermal stability and solubility, thus was potential in generating high-performance keratin materials from chicken feather. The study investigated the ultrasonic effect on feather keratin systemically and proposed a green alternative strategy for keratin regeneration, which might be meaningful for utilizing and valorizing feather waste.

## CRediT authorship contribution statement

**Xiaojie Qin:** Investigation, Formal analysis, Writing – original draft. **Chuan Yang:** Investigation, Data curation. **Yujie Guo:** Methodology, Investigation. **Jiqian Liu:** Writing – review & editing. **Johannes H. Bitter:** Supervision, Methodology. **Elinor L. Scott:** Supervision, Methodology. **Chunhui Zhang:** Supervision, Project administration, Funding acquisition.

## Declaration of Competing Interest

The authors declare that they have no known competing financial interests or personal relationships that could have appeared to influence the work reported in this paper.

## Appendix A Supplementary data

Supplementary Table S1 Conditions of Box-Behnken experiment design. Note: The optimal conditions of the single-factor experiment were 200 W, 4 h, and 15% of Cys.

Supplementary Table S2 The crystallinity index of regenerated keratin.

Supplementary Table S3 ANOVA analysis for response surface quadratic model of variance. Note: A represents ultrasonic power, B represents ultrasonic time, and C represents Cys fraction.
