# Supplementary material for: Effect of ultrasound on keratin valorization from chicken feather waste: Process optimization and keratin characterization
Source: Ultrason Sonochem. 2023 Jan 10;93:106297. doi: 10.1016/j.ultsonch.2023.106297 (PMC9860336; doi:10.1016/j.ultsonch.2023.106297)
Supplement: Supplementary Table S1 — Conditions of Box-Behnken experiment design. Note: The optimal conditions of the single-factor experiment were 200 W, 4 h, and 15% of Cys. [file mmc1.docx]

**Table S1**

| **Factors** | **Levels** | | |
| --- | --- | --- | --- |
|  | -1 | 0 | 1 |
| **Ultrasonic power (A/W)** | 100 | 200 | 300 |
| **Ultrasonic time (B/h)** | 2 | 4 | 6 |
| **Cys fraction (C/%)** | 10 | 15 | 20 |
