# Supplementary material for: Effect of ultrasound on keratin valorization from chicken feather waste: Process optimization and keratin characterization
Source: Ultrason Sonochem. 2023 Jan 10;93:106297. doi: 10.1016/j.ultsonch.2023.106297 (PMC9860336; doi:10.1016/j.ultsonch.2023.106297)
Supplement: Supplementary Table S2 — The crystallinity index of regenerated keratin. [file mmc2.docx]

**Table S2**

| **Time (h)** | **2** | **4** | **6** | **8** | **10** |  |
| --- | --- | --- | --- | --- | --- | --- |
| **CI (%)** | 61.93 | 61.92 | 61.61 | 61.67 | 63.16 |  |
| **Power (W)** | **100** | **200** | **300** | **400** | **500** | **600** |
| **CI (%)** | 62.27 | 62.77 | 62.71 | 61.91 | 62.09 | 61.54 |
