# Supplementary material for: Effect of ultrasound on keratin valorization from chicken feather waste: Process optimization and keratin characterization
Source: Ultrason Sonochem. 2023 Jan 10;93:106297. doi: 10.1016/j.ultsonch.2023.106297 (PMC9860336; doi:10.1016/j.ultsonch.2023.106297)
Supplement: Supplementary Table S3 — ANOVA analysis for response surface quadratic model of variance. Note: A represents ultrasonic power, B represents ultrasonic time, and C represents Cys fraction. [file mmc3.docx]

**Table S3**

| **Sources of variance** | **Sum of squares** | **df** | **Mean square** | **F value** | **P value**  **（P > F）** |
| --- | --- | --- | --- | --- | --- |
| **Model** | **42577.31** | **9** | **4730.81** | **17.97** | **0.0005**** |
| **A** | **2997.70** | **1** | **2997.70** | **11.39** | **0.0118*** |
| **B** | **17491.05** | **1** | **17491.05** | **66.45** | **0.0001**** |
| **C** | **3936.95** | **1** | **3936.95** | **14.96** | **0.0062*** |
| **AB** | **176.36** | **1** | **176.36** | **0.67** | **0.4400** |
| **AC** | **141.85** | **1** | **141.85** | **0.54** | **0.4867** |
| **BC** | **548.26** | **1** | **548.26** | **2.08** | **0.1922** |
| **A^2^** | **2749.02** | **1** | **2749.02** | **10.44** | **0.0144*** |
| **B^2^** | **9804.10** | **1** | **9804.10** | **37.25** | **0.0005*** |
| **C^2^** | **3114.94** | **1** | **3114.94** | **11.83** | **0.0108*** |
| **Residual** | **1842.53** | **7** | **263.22** |  |  |
| **Lack of fit** | **1476.66** | **3** | **492.22** | **5.38** | **0.0688** |
| **Pure error** | **365.86** | **4** | **91.47** |  |  |
| **Cor total** | **44419.84** | **16** |  |  |  |
| **Coefficient** | **R^2^=0.9585 Adj R^2^=0.9052 Pre R^2^=0.4552 Adeq precision=12.196** | | | | |

Note: A represents ultrasonic power, B represents ultrasonic time, and C represents Cys fraction.
